# Department of Surgery

**Published:** 1900-12

**Authors:** W. E. A. Wyman, J. A. Sloan, Robert Jay

**Affiliations:** Burlington, Wis.; San Francisco, Cal.; West Liberty, Ia.


### DEPARTMENT OF SURGERY.

By W. E. A. Wyman, M.D.V., V.S.,
BURLINGTON, WIS.

J. A. Sloan, B.Sc., M.D.V.,
SAN FRANCISCO, CAL.

Robert Jay, M.D.V.,

WEST LIBERTY, IA.

### Hoof Diseases.

(Continued from page 686.)

To appreciate thoroughly the various pathological states and properly treat them, at least, a fair working knowledge of the anatomo-histological arrangements of the horn-producing parts is essential. The continuation of the external skin enclosed by the hoof is termed pododerm; it secretes the hoof. In it three layers are recognized: 1. A modified epidermis. 2. Cutis. 3. Subcutis.

The modified epidermis is represented by the horny capsule, the hoof.

The cutis or pododerm exhibits: 1. The perioplic band. 2. Coronary band. 3. Podophyllous tissue. 4. Fleshy sole. 5. Fleshy frog.

The perioplic band lies between the external skin and coronary band. It is of granular, velvety appearance; its surface is densely studded with delicate villi. Its physiological function consists in the formation of the periople or outer protective layer.

The coronary band lies between the perioplic band and sensitive laminæ; at the heels it turns in, losing itself in the fleshy sole or meeting the other half about at the point of the frog. Its surface also has a granular, velvety look, due to many fine papillæ, equipped with the secondary lamellæ, same as those of the fleshy sole. Its physiological function is the formation of the main mass of the hoof—the middle layer and the bars. Next comes the fleshy wall, rich in bloodvessels, covering the anterior face of the os pedis and lower portion of the lateral cartilages; turning in at the heels it runs forward between the bars, producing part of the coronary band and fleshy sole, forming near the middle of the sole the bar laminæ.

There is no subcutis where the fleshy wall covers the os pedis, both being directly and intimately united. In contrast to other parts the podophyllous tissue is made up of parallel leaves running from above to below, separated by grooves into which the horny laminæ fit. As the fleshy leaves merge into the sole, their laminated appearance loses itself, and papillæ are seen instead. Each fleshy leaf shows collateral leaves, designated respectively as primary and secondary laminæ. The physiological function of the fleshy wall lies in the production of the keraphyllous tissue, uniting the pododerm and horny wall.

The fleshy sole covers the solar surface of the os pedis, replacing, so to speak, the periosteum. Its velvety appearance also is due to villi of similar size and make-up as those of the coronary band. It forms the horny sole.

The fleshy frog, covering the plantar cushion, exhibits shorter and thinner villi with collateral lamellæ. Its function is the formation of the horny frog.

The extent of an at least modified subcutis is very limited; underneath the coronary band it braces the many bloodvessels; under the upper margin of the fleshy wall it covers the extensor pedis tendon and lateral cartilages, being represented by the plantar cushion below.

This coarse, anatomical sketch having been deemed necessary, some of the leading histological features of the cutis are desirable.

A cross section of the fleshy wall, according to Möller, exhibits the periosteal layer (stratum periostale), vascular layer (stratum vasculosum), leaf-like layer (stratum phyllodes), and rete Malpighii.

The innermost layer of the stratum periostale is only represented where the podophyllous tissue is in immediate contact with the os pedis and lateral cartilages. Right here it is also termed parachondrial layer (stratum parachondrale). The periosteal layer, which is at the same time the periosteum of the os pedis, has primarily a connective tissue composition, exhibiting less vessels and nerves than the vascular layer. Next to it, composed of connective tissue and elastic fibres with many nerves, bloodvessels, and lymph-vessels, comes the vascular layer. Above it the phylloid layer with its many laminæ; each lamina, as previously stated, has numerous collateral leaves, the whole being made up of strong connective tissue enclosing the bloodvessels.

The rete Malpighii, composed of cells only, and forming the horn cells, covers the entire surface of the phylloid layer.

The fleshy sole, as already stated, exhibits villi, sections showing them to be of similar structure as the laminæ of the fleshy wall. The same refers to the coronary band, but the periosteal layer is absent, being replaced by a subcutis.

The fleshy frog also has no periosteal layer; the vascular layer is less vascular, while an intimate union exists between it and the plantar cushion. It is also the only part of the pododerm containing sweat glands, first described by Ercolani, 1861. These glands are also capable of producing an oily substance. (Lungwitz, Eberlein, Möller.)

*Wounds.* Wounds of the pododerm, same as in other parts, may be contused, incised, lacerated, etc.; superficial or deep, aseptic, infected, bleeding, granulating, and so on, while thermic and chemical agents may finally exert detrimental influences.

According to locality, the coronary band, podophyllous tissue, velvety tissue of sole or frog, may be involved. Contusions and punctures of the pododerm are most frequently met. During slippery weather, wounds about the coronary region from sharp shoes are common. Horses which interfere on account of faulty positions of their legs (toe-out, for instance), injure that locality; the region of the heels is often bruised and incised in those which overreach or forge. Finally, kickers may drive splinters into these parts, and animals working about the public dumping places are mainly exposed to cuts from glass, tin cans, wire, etc.

The fleshy wall is exposed to nail-pricks (in shoeing), while penetrating seams of the horny box may pinch the sensitive laminæ. Excessive paring of so-called corns often leads to trouble.

The fleshy sole and frog are mainly injured by punctures and bruises from nails, stones, glass, etc. Burns from acids, alkalies, the actions of fire and cold are also possibilities.

The ordinary symptoms, such as hemorrhage, gaping of wounds, necessarily depend on the parts involved. Thus wounds of the coronary band, unless the diameter of the offending agent is small, bleed freely, especially when weight is borne by the foot, stopping more or less as frog pressure is removed. The presence of a rich nerve-supply explains the decided pain accompanying wounds of the pododerm, increased by subsequent inflammation and expressed by a supporting leg lameness plus increased pulsation in the arterial blood-supply to the parts. Depending on the extent of the lesion decrease or even suspension of appetite with slight aseptic or even infectious fever may be present.

As a rule, no difficulties are experienced in making a diagnosis. The history of the case, increased pulsation of the digital arteries, supporting leg lameness, pain on palpation, if necessary employing the searching knife to locate the point of entrance, as the horny cells by swelling hide it.

The greatest difficulty is found in estimating the depth of the fresh wound, as but few animals will permit the proper use of the sound on account of the terrible pain; to be sure, probing in such cases is of doubtful value when such vital parts as the neighborhood of the perforans tendon, lateral cartilage, plantar cushion, or pedal articulation are involved, simply by reason of a possible infection. Such wounds almost invariably at the very outset are infected, and the fact that such a wound healed by first intention is no proof of a non-infection, but rather explained by the cleansing influence of a free hemorrhage. Therefore, a doubtful prognosis is generally indicated, as tetanus, malignant œdema, the location of the wound and complications, as quittors, necrosis of the perforans tendon, suppurating pododermatitis, etc., are to be thought of.

Antiseptic wound treatment is the *conditio sine qua non*. After the edges of the wound have been trimmed, foreign bodies removed, the parts are thoroughly irrigated with a 1 per cent. solution of formalin or 1 per cent. solution of corrosive sublimate; when gaping is marked a few stitches will materially shorten the period of healing. If possible, a dressing is applied which is kept moist with warm antiseptics, the writer preferring 1 per cent. solution of formalin or 1 per cent. solution of corrosive sublimate.

Unless the animal shows pain and fever (above 101.5° to 102° F.) it is well to leave the warm, moist dressing on for two or three days. In those cases where necrosis of tissue is reasonably expected (bruised wounds) the warm, moist dressing, changed one or more times daily, according to the case, is continued until the necrosed parts are expelled; in this way demarcation is favorably influenced. In cases where no visible necrosis is present, in those where no necrosis is expected, and, finally, in those where the necrotic zones have been removed, my favorite dressing consists of Merck's tannoform changed daily until the parts are dry and covered with a brown coat, which is left undisturbed. Should granulations become luxuriant a pressure dressing of equal parts of alum and tannoform is applied. Wherever pus is freely discharged you will find that a saturated solution of tannoform in alcohol injected into the canal or applied to the pus-producing surface but a few times will surprise you. The writer can safely say that Merck's tannoform is of great value both externally and internally. Use it, get acquainted with it, study its innumerable possibilities, ask no impossibilities, and you will soon learn it deserves your full confidence, as it is reliable and cheap. Animals which have to go to work before cicatrization is complete are given a permanent pix liquida bandage.

Wounds which penetrate into the sole or frog must be given free drainage by a free removal of the horny sole or frog. This is followed by a disinfection of a 1 per cent. formalin solution, and this by the alcohol-tannoform solution. A protective warm and moist packing (I usually take cotton-waste) is now put on, and the animal seen in twenty-four to forty-eight hours, as the case may be. Should the owner insist on working the animal at once the ordinary splint dressing is applied to the shoe.

When the pain increases, showing that the pathological lesions are progressing, the writer usually confines the animal at once, splits the infected tract, and, if necessary, curettes it. This is followed by a formalin irrigation, 1 per cent., and wherever practicable a tannoform bougie is introduced every other to every third day. Merck gives the following formula for five bougies, two inches long, one-fifth to three-fifth inches thick: Tannoform, 75 grains; cocoa butter, 375 grains; castor oil, q. s.—M. Of course, absolute drainage is essential. An occasional hot antiseptic foot bath is a valuable therapeutic adjunct.

---

Among the visitors to the National Horseshow in Madison Square Garden in November were to be noted Drs. R. S. Huidekoper, of Washington, D.C.; Wm. Jakeman, of Halifax, N. S.; H. D. Gill and M. O. M. Knott, of New York City, N. Y.; W. Horace Hoskins, of Philadelphia, Pa.; Wm. Herbert Lowe, of Paterson, N. J., and T. Earle Budd, of Orange, N. J.

Dr. T. A. Geddes, of the Bureau of Animal Industry, has been sent to England and the Continent, where he will apply the tuberculin-test to all cattle and breeding stock destined for shipment to the States. This will save much annoyance incidental to shipping, and prevent the reshipment of those condemned on examination on this side.

Dr. G. Howard Davison, of Millbrook, N. Y., was the winner of the first prize with his Shropshire sheep at the International Live-stock Exposition at Chicago in November.

Dr. E. A. A. Grange has severed his connection with the Humane Society of New York, and is temporarily caring for the practice of Dr. H. A. Paget, of Scranton, Pa.

